# Dynamic assessment of venous thromboembolism risk in patients with cancer by longitudinal D‐Dimer analysis: A prospective study

**DOI:** 10.1111/jth.14774

**Published:** 2020-04-15

**Authors:** Florian Posch, Julia Riedl, Eva‐Maria Reitter, Michael J. Crowther, Ella Grilz, Peter Quehenberger, Bernd Jilma, Ingrid Pabinger, Cihan Ay

**Affiliations:** ^1^ Clinical Division of Haematology and Haemostaseology Department of Medicine I Medical University of Vienna Vienna Austria; ^2^ Division of Oncology Department of Internal Medicine Medical University of Graz Graz Austria; ^3^ Center for Biomarker Research in Medicine (CBmed Ges.m.b.H.) Graz Austria; ^4^ Department of Health Sciences Centre for Medicine University of Leicester Leicester UK; ^5^ Department of Anesthesia and Critical Care SMZ Ost – Danube Hospital Vienna Austria; ^6^ Department of Laboratory Medicine Medical University of Vienna Vienna Austria; ^7^ Section of Hematology & Immunology Department of Clinical Pharmacology Medical University of Vienna Vienna Austria; ^8^ I.M. Sechenov Fist Moscow State Medical University (Sechenov University) Moscow Russia

**Keywords:** cancer, venous thromboembolism, D‐dimer, joint model, longitudinal biomarker analysis

## Abstract

**Background:**

Venous thromboembolism (VTE) is a frequent complication of cancer. Elevated D‐dimer is associated with an increased risk of cancer‐associated VTE. Whether changes in D‐dimer over time harbor additional prognostic information that may be exploited clinically for dynamic prediction of VTE is unclear.

**Objectives:**

To explore the potential role of longitudinal D‐dimer trajectories for personalized prediction of cancer‐associated VTE.

**Patients/Methods:**

A total of 167 patients with active malignancy were prospectively enrolled (gastrointestinal: n = 59 [35%], lung: n = 56 [34%], brain: n = 50 [30%], others: n = 2 [1%]; metastatic disease: n = 74 [44%]). D‐dimer (median = 0.8 µg/mL [25th‐75th percentile: 0.4‐2.0]) was measured at baseline and during 602 monthly follow‐up visits. Joint models of longitudinal and time‐to‐event data were implemented to quantify the association between D‐dimer trajectories and prospective risk of VTE.

**Results:**

VTE occurred in 20 patients (250‐day VTE risk = 12.1%, 95% confidence interval [CI], 7.8‐18.5). D‐dimer increased by 34%/month (0.47 µg/mL/month, 95% CI, 0.22‐0.72, *P* < .0001) in patients who developed VTE, but remained constant in patients who did not develop VTE (change/month = −0.06 µg/mL, 95% CI, −0.15 to 0.02, *P* = .121). In joint modeling, a doubling of the D‐dimer trajectory was associated with a 2.8‐fold increase in the risk of VTE (hazard ratio = 2.78, 95% CI, 1.69‐4.58, *P* < .0001). This finding was independent of established VTE risk factors. Highly personalized, dynamic predictions of VTE conditional on individual patients’ D‐dimer trajectories could be obtained.

**Conclusions:**

D‐dimer increases before the onset of cancer‐associated VTE, but remains constant over time in patients without VTE. This study represents proof‐of‐concept that longitudinal trajectories of D‐Dimer may advance the personalized assessment of VTE risk in the oncologic setting.

**Essentials**

- Whether longitudinal measurement of D‐Dimer improves VTE risk stratification in patients with cancer is unclear.
- We modelled the association between D‐Dimer trajectories and risk of cancer‐associated VTE.
- D‐Dimer increased before the onset of cancer‐associated VTE.
- D‐Dimer remained constant over time in patients without VTE.
- Highly personalized dynamic predictions of VTE based on D‐Dimer trajectories could be obtained.

## 1 INTRODUCTION

Cancer and coagulation are highly linked processes.^1^ Although an activated coagulation cascade contributes to tumor progression and metastasis, cancers induce a hypercoagulable state that promotes venous thromboembolism (VTE).^2^ VTE is a frequent complication and a leading cause of morbidity and death in patients with cancer.^3^ The overall risk of developing VTE in patients with active malignancy is approximately 5% to 10% over 2 years,^4^ but strongly varies between patient subgroups according to prognostic factors such as tumor type.^5^ Indeed, 2‐year VTE risks can be as low as 2% in patients with prostate cancer and as high as 30% in patients with upper gastrointestinal malignancies.^6^ Randomized controlled trials have shown that prophylactic anticoagulation can significantly reduce the risk of VTE in the oncologic setting, but only patients at very high risk derive a clinically meaningful magnitude of benefit from this intervention.^7^ Thus, the identification of cancer patients at high risk of VTE is an important area of clinical research and necessary prerequisite for improving the therapeutic ratio of prophylactic anticoagulation in the oncologic setting. Several clinical prediction models currently exist for VTE risk assessment in cancer patients, including the Khorana score,^6^ but all of these models appear to have modest prognostic performance^8^, ^9^, ^10^. During the past several years, we and others have shown that elevated biomarkers of hypercoagulability, such as D‐dimer, are strongly associated with a higher risk of VTE,^11^, ^12^ and can furthermore improve established clinical prediction models. This supports the concept that hemostatic biomarkers could support physicians in selecting patients with the highest VTE risk for prophylactic anticoagulation while sparing low‐VTE‐risk patients from unnecessary burden and bleeding complications.^13^

However, coagulation and cancer are dynamic processes that may be influenced by patient‐ and treatment‐related factors that change over time, such as disease progression and anti‐neoplastic therapies. Indeed, cancer nowadays represents an increasingly chronic disease, and hemostatic biomarker levels may thus significantly change during the patient journey. A single hemostatic biomarker measurement for VTE risk prediction may thus represent only one “snapshot” of cancer‐associated hypercoagulability at a single point in time. All currently available biomarkers and clinical prediction models for cancer‐associated VTE were developed as a single measurement at a baseline time point.^8^ Whether repeated quantification of hemostatic biomarkers over time may represent a clinically superior approach for VTE risk stratification in cancer patients is unclear.

Thus, we hypothesize that longitudinal trajectories of the hemostatic biomarker D‐dimer may harbor important “dynamic” prognostic information on cancer‐associated VTE risk beyond a single D‐dimer measurement in time, and may improve the clinical assessment of venous thromboembolic risk in patients with cancer. So‐called joint models of longitudinal and time‐to‐event data have been developed for examining this hypothesis.^14^, ^15^, ^16^ In this prospective cohort study, we used joint models to define the role of the longitudinal D‐dimer trajectory for dynamic prediction of VTE in patients with cancer, with the aim of answering whether such a concept could be a clinically meaningful strategy for improving VTE risk assessment in oncology.

## 2 METHODS

### 2.1 Study design

In this longitudinal substudy of the prospective and ongoing Vienna Cancer and Thrombosis Study (CATS), we enrolled patients with solid cancers who were treated at Vienna General Hospital from January 2011 to July 2014. Detailed in‐ and exclusion criteria were reported previously.^17^, ^18^ Briefly, eligible patients had histologically confirmed, newly diagnosed, or relapsed active cancer, were not undergoing anti‐neoplastic therapy at the baseline date, and did not receive anticoagulation (except for low‐dose aspirin and prophylactic‐dose low‐molecular‐weight heparins during inpatient stays). Patients were followed with repeated visits each approximately 1 month apart, for a maximum duration of 250 days and six follow‐up visits. The primary endpoint of this analysis was a composite of symptomatic and objectively confirmed deep vein thrombosis (DVT) and/or pulmonary embolism (PE) during the 250‐day observation period. All events were adjudicated by an independent panel (n = 3 experts in vascular medicine, radiology, and nuclear medicine). So‐called incidental PE was counted as an event if the panel deemed it to be of clinical significance requiring anticoagulation. Fatal PE was defined as (1) PE as the cause of death on autopsy record or (2) assessment of PE as the immediate cause of death by the adjudication committee.

### 2.2 Laboratory analysis

Citrated venous blood samples (3.2% trisodium citrate tube, VACUETTE®, Greiner‐Bio One) were obtained at each visit by antecubital venipuncture or from central venous catheters. D‐dimer was assessed within our hospital's routine laboratory with immuno‐turbidimetry using the STalia D‐DI assay (Diagnostica Stago).^19^

### 2.3 Statistical methods

All statistical analyses were performed with Stata 15.1 (Stata Corp.). Cumulative VTE incidence was estimated with competing risk estimators, treating death from any cause other than fatal VTE as the competing event.^20^, ^21^, ^22^, ^23^ The association between baseline D‐dimer and VTE was modeled with uni‐ and multivariable Weibull proportional hazards regression.^24^ The primary analysis quantity of this study was the association parameter α (i.e., the association between the longitudinal D‐dimer biomarker trajectory and the hazard of VTE expressed as a hazard ratio).^16^ α was estimated with a joint model for longitudinal and time‐to‐event data,^25^ which consist of a longitudinal component (here: the D‐dimer trajectory) and a time‐to‐event component (here: the hazard of VTE). These two components are linked together via α. The joint model was specified as follows: random‐intercept‐and‐slope model with a random effect of linear follow‐up time for the longitudinal component (because nonlinear time specifications did not provide a better fit to the data (Akaike information criterion [AIC]: 3308 [linear time] vs 3310 [linear + squared + cubic time]); Weibull proportional hazards regression model for the time‐to‐event component; a “current association” specification of α,^26^ exclusion of one patient with D‐dimer levels > 40 µg/mL that prevented model convergence; and an unstructured variance‐covariance‐matrix allowing the two random effects (intercept and slope) to be correlated. Multivariable analysis included time‐invariant baseline covariates (such as tumor type) in both the longitudinal and time‐to‐event submodels. Moreover, we adjusted α for whether patients had metastatic cancer at baseline, and investigated a “first derivative” specification of α (i.e., the “slope” or rate of change in D‐dimer).^26^ All joint models were fitted with the user‐contributed Stata routine stjm,^27^ freely available on the Boston College Statistical Software Components archive.^28^ Two specifications of D‐dimer were studied in the joint models, namely D‐dimer on a continuous original scale (µg/dL) and on a log2‐transformed scale (i.e., per doubling). The fit of these two specifications was compared using the AIC. Predictions of VTE risk for individual patients conditional on their D‐dimer trajectories were obtained using a Stata routine (stjmcsurv, currently in development but freely available online)^29^ based on the dynamic prediction approach of Rizopoulos.^30^ A Strengthening The Reporting of Observational studies in Epidemiology checklist (see supporting information). The full analysis code is available on requested from F.P.

## 3 RESULTS

### 3.1 Analysis at baseline

One‐hundred and sixty‐seven patients with a median age of 63 years [25th‐75th percentile: 53‐69] were included (Table 1). The most frequent tumor types were lung cancers (n = 56), primary brain tumors (n = 50), and pancreatic adenocarcinomas (n = 34). Most patients suffered from metastatic disease (n = 74, 44%), whereas the remaining patients had localized (n = 57, 34%) or locally advanced (n = 36, 22%) cancers, respectively.

**Table 1:** Baseline characteristics of the study population (n = 167)

Variable	n (% miss.)	Overall (n = 167)	No VTE during follow‐up (n = 147)	VTE during follow‐up (n = 20)	*P* ^a^
Demographic characteristics					
Age at entry (years)	167 (0%)	62.7 [53.1‐69.0]	62.7 [53.3‐68.8]	58.8 [52.1‐71.5]	.894
Female	167 (0%)	73 (44%)	63 (43%)	10 (50%)	.546
BMI (kg/m^2^)	167 (0%)	24.5 [21.6‐27.7]	24.2 [21.2‐27.5]	25.6 [23.4‐30.3]	.049
Tumor characteristics					
Type	167 (0%)	/	/	/	.056
Lung	/	56 (34%)	53 (36%)	3 (15%)	/
Brain	/	50 (30%)	45 (31%)	5 (25%)	/
Pancreas	/	34 (20%)	24 (16%)	10 (50%)	/
Colorectal	/	22 (13%)	20 (14%)	2 (10%)	/
Stomach	/	3 (2%)	3 (2%)	0 (0%)	/
Breast	/	1 (1%)	1 (1%)	0 (0%)	/
Others (Malignant pleural mesothelioma)	/	1 (1%)	1 (1%)	0 (0%)	/
Newly diagnosed malignancy	167 (0%)	159 (95%)	140 (95%)	19 (95%)	.999
Tumor stage^b^	167 (0%)	/	/	/	.952
Local (TNM N0 M0)	/	57 (34%)	51 (35%)	6 (30%)	/
Locally advanced (TNM N + M0)	/	36 (22%)	32 (22%)	4 (20%)	/
Metastatic (TNM M1)	/	74 (44%)	64 (44%)	10 (50%)	/
Khorana score and its items^c^					
Khorana score (points)	167 (0%)	2 [1‐2]	2 [1‐2]	2 [1.5‐2]	.676
Khorana score high (≥3 points)	167 (0%)	34 (20%)	31 (21%)	3 (15%)	.768
Low/moderate VTE risk tumor sites	167 (0%)	23 (14%)	21 (14%)	2 (10%)	.084
High VTE risk tumor sites	167 (0%)	57 (34%)	54 (37%)	3 (15%)	/
Very high VTE risk tumor sites	167 (0%)	87 (52%)	72 (49%)	15 (75%)	/
Hemoglobin < 10 g/dL and/or ESA use	167 (0%)	2 (1%)	2 (1%)	0 (0%)	.999
White blood count ≥ 11 G/L	167 (0%)	26 (16%)	24 (16%)	2 (10%)	.743
Platelet count ≥ 350 G/L	167 (0%)	29 (17%)	29 (20%)	0 (0%)	**.026**
BMI ≥ 35kg/m^2^	167 (0%)	3 (2%)	2 (1%)	1 (5%)	.320
Hemostatic biomarkers					
D‐dimer at baseline (µg/mL)	165 (1%)	0.97 [0.54‐2.05]	0.91 [0.51‐1.96]	1.88 [1.09‐6.04]	**.003**

Note

Distribution overall as well as by prospective VTE status. Continuous variables are reported as medians [25th‐75th] percentile, and count data as absolute frequencies (%). n (%miss.) reports the number of patients with fully observed data (% missing).

Abbreviations: BMI, Body Mass Index; ESA, Erythropoiesis‐stimulating agents; TNM, Tumor Node Metastasis classification; VTE, Venous thromboembolism.

^a^ *P* values were derived using Wilcoxon's rank‐sum, χ^2^, or Fisher's exact tests (*P* values ≤ .05 are reported in bold font).

^b^ Patients with primary brain tumors were assigned to the “local” stage group.

^c^ Tumor site categories were defined as in the original publication by Khorana et al (i.e., colorectal cancer included in the “low/moderate VTE risk” group),^6^ with brain tumors being assigned to the “very high VTE risk” group according to Ay et al.^37^

### 3.2 Analysis of VTE risk and its baseline predictors

During the observation period of 250 days, 20 patients developed VTE and 34 patients died. This corresponded to a cumulative 250‐day VTE risks of 12.1% (95% confidence interval [CI], 7.7‐17.6) with competing risk analysis and 13.1% (95% CI, 8.6‐19.5) with 1‐Kaplan‐Meier analysis, respectively (Figures S1 and S2). VTE event types were lower extremity DVT (n = 8, 40%), PE (n = 7, 35%), fatal PE (n = 2, 10%), lower extremity DVT + PE (n = 1, 5%), lower extremity DVT + portal vein thrombosis (n = 1, 5%), and inferior vena cava thrombosis (n = 1, 5%), respectively. In univariable Weibull regression of baseline variables as predictors of VTE risk, only higher body mass index (hazard ratio [HR] per 5 kg/m^2^ increase = 1.61, 95% CI, 1.11‐2.31, *P* = .011) and elevated D‐Dimer (HR per doubling = 1.73, 1.32‐2.27, *P* < .0001) were associated with a higher risk of VTE (Table S1).

### 3.3 Evolution of D‐dimer levels over time in patients who did and did not develop VTE

**Figure 1:**
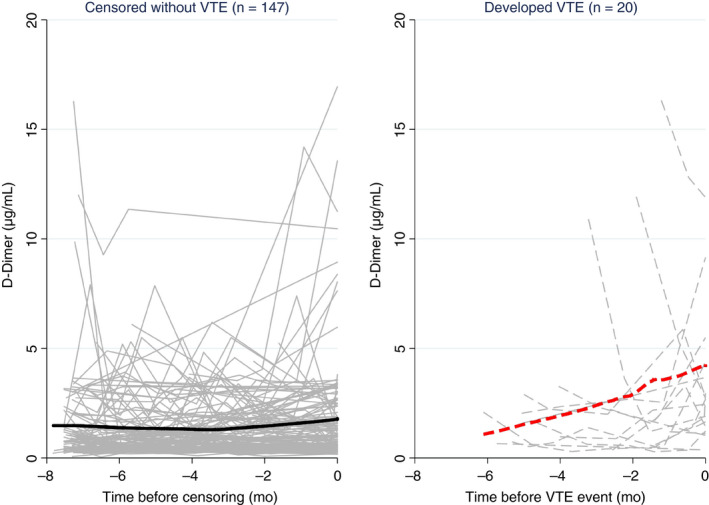
Line plot of D‐dimer trajectories in patients who did (right, gray dashed lines) and did not (left, gray solid lines) develop VTE during follow‐up. Each line represents the D‐dimer trajectory of a single patient. The bold solid line (left) and bold dashed line (right) represent moving averages (locally weighted sum of squares [LOWESS] nonparametric smoother). Although D‐dimer remained relatively constant over time in patients who did not develop VTE, it increased steadily in patients before the onset of VTE. Note that the time on the x‐axis of both panels is inverted (i.e., it represents the time *before* the onset of VTE or censoring without VTE).

After baseline, patients returned for 602 follow‐up visits, for a total number of 769 visits included in the analysis (median number of visits per patient = 5, 25th‐75th percentile: 3‐7, range: 1‐7). Measurements of D‐dimer were available for 761 visits (1% missing, Table S2). In univariable joint modeling of D‐dimer and time‐to‐thrombosis, mean D‐dimer at baseline was 1.84 µg/mL and remained constant during follow‐up in the overall study population (change = −0.03µg/mL/month, *P* = .573, Table 2). Notably, the change in D‐dimer over time was different in patients who did and did not develop VTE during follow‐up (Figure 1). In detail, D‐dimer remained stable over time in patients who did not develop VTE, but increased by 0.47 µg/mL/month in patients who developed VTE (*P* < .0001, Table 2). This result could be confirmed on a relative scale, where D‐dimer decreased by 2.6%/month in patients who did not develop VTE and increased by 34%/month in patients who developed VTE, respectively (*P* < .0001, Table 2).

**Table 2:** Change in D‐dimer over time: distribution overall and by prospective VTE event status

D‐dimer scale	D‐dimer change over time (95% CI, *P*)			
	All patients (n = 167)	No VTE event (n = 147)	VTE (n = 20)	Difference
D‐dimer change (µg/mL/month from baseline)	−0.03 µg/mL/month (−0.12‐0.06, *P* = .573)	−0.06 µg/mL/month (−0.15‐0.02, *P* = .121)	+0.47 µg/mL/month (0.22‐0.72, *P* < .0001)	0.53 µg/mL/month (0.28‐0.78, *P* < .0001)
D‐dimer change (%/month from baseline)	−0.3%/month (−3.3‐2.7, *P* = .846)	−2.6%/month (−5.30.1, *P* = .061)	+33.7%/month (23.4‐44.8, *P* < .0001)	N/A (*P* < .0001)

Note

Results are from the longitudinal component of joint models without other covariables. Changes in % were obtained by using log‐transformed D‐dimer.

Abbreviations: 95% CI, 95% confidence interval; *P*, Wald‐test *P* value.

### 3.4 Longitudinal D‐dimer trajectories for prediction of VTE risk

In joint modeling of longitudinal D‐dimer trajectories and time to VTE, patients with an elevated D‐dimer over time experienced a higher risk of VTE (HR per doubling of D‐dimer at any time of follow‐up (i.e., the association parameter α) = 2.78 (95% CI, 1.69‐4.58, *P* < .0001), models 1 and 2 in Table ^3^). This prognostic association applied similarly to patients with and without metastatic cancers (Table S3), and prevailed both with respect to magnitude and strength of association upon multivariable adjustment for the Khorana score in both the longitudinal and survival submodels (adjusted HR for the D‐dimer trajectory‐VTE association = 1.45, 95% CI: 1.25‐1.69, *P* < .0001, Table S4). A sensitivity analysis with an additional first derivative specification of the association parameter α did not suggest that a higher rate of D‐dimer increase provides additional prognostic information on VTE risk beyond the usual longitudinal D‐dimer trajectory (*P* = .175).

**Table 3:** Associations of longitudinal D‐dimer trajectories and prospective thrombotic risk–univariable joint models

Univariable joint model	Trajectory variable	Association parameter α	95%CI	*P*
Model 1	D‐dimer (per 1 µg/mL increase)	1.46	1.25‐1.69	<.0001
Model 2	D‐dimer (per doubling)	2.78	1.69‐4.58	<.0001

Note

Hazard ratios per doubling were obtained by using log2‐transformed D‐dimer. Log2‐transformed D‐dimer gave a better fit to the data than D‐dimer on its original µg/mL scale (AIC: 2434 vs 3308).

Abbreviations: 95% CI, 95% confidence interval; AIC, Akaike information criterion; *P*, Wald‐test *P* value.

### 3.5 Personalized prediction of VTE according to D‐dimer trajectories

**Figure 2:**
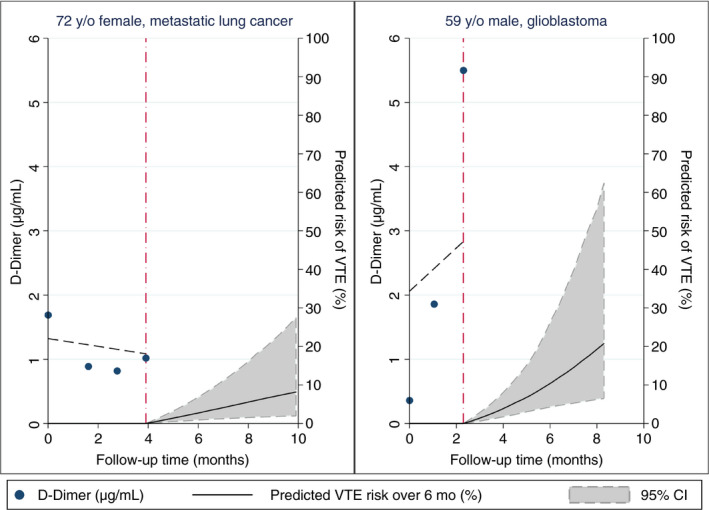
Personalized predictions of 6‐month VTE risk according to two study patients’ individual D‐dimer trajectories. Predictions are based on model 1 in Table 3 (i.e., the prediction are only based on the D‐dimer trajectory and not on covariables such as tumor type). Left, The patient is a 72‐year‐old lady with metastatic lung cancer. During her four study visits, D‐dimer levels remained relatively stable over time. According to the model, her 6‐month predicted VTE risk (following her last visit approximately 4 months after study inclusion, red vertical dash‐dotted line) is below 10%. This patient did not develop VTE during 8 months of follow‐up. Right, The second patient is a 59‐year‐old man with glioblastoma and strongly increasing D‐dimer levels over his three study visits. According to the model, his 6‐month predicted VTE risk (following his last visit approximately 2 months after study inclusion, red dash‐dotted line) is above 20%. Note that this patient's 95% confidence interval for the VTE risk prediction is wider than the corresponding confidence interval from the patient in the left panel due to fewer visits and follow‐up time. This patient developed symptomatic lower‐extremity DVT six weeks after his last visit.

The joint model (model 1 in Table 3) could be used to obtain highly personalized predictions of VTE conditional on each patient's individual D‐dimer trajectory. To illustrate this finding, Figure 2 shows examples of dynamically assessed VTE risks for two individual patients. A 72‐year‐old woman with metastatic lung cancer returned for three follow‐up study visits (Figure 2, left). Her D‐dimer measurements were always elevated but remained relatively stable over time, and the model estimated a 6‐month VTE risk below 10% following her last study visit approximately 4 months after baseline. The woman did not develop VTE. A 59‐year‐old male with glioblastoma returned for two study visits after baseline (Figure 2, right). His baseline D‐dimer was within the normal range, but strongly increased during follow‐up. The model estimated a 6‐month VTE risk above 20% following his last study visit approximately 2.5 months after baseline. The patient subsequently developed a pelvic‐vein DVT.

## 4 DISCUSSION

In this longitudinal substudy of the prospective Vienna Cancer and Thrombosis Study, we have quantified changes of D‐dimer over time and estimated the relationship of these changes with the risk of cancer‐associated VTE using so‐called joint models. Analyzing more than 700 study visits from 167 patients with solid cancers, we observed that D‐dimer levels increased before the onset of cancer‐associated VTE, but remained constant over time in patients who did not develop VTE. This longitudinal D‐dimer trajectory harbored important time‐dependent information on the occurrence of VTE and could be used to obtain highly personalized “dynamic” predictions of VTE. These results represent a proof‐of‐concept that the consideration of longitudinal D‐dimer trajectories, beyond their levels at a single baseline point in time, may advance the personalized assessment of VTE risk in the oncologic setting.

Cancer is becoming an increasingly chronic disease.^31^, ^32^ Consequently, patient‐related, tumor‐related, and treatment‐related risk factors can change over the patient journey and dynamically modify an individual patient's prognosis with regard to VTE and survival.^5^ The dynamic reassessment of prognosis according to changes in clinical and laboratory parameters is a highly intuitive concept for clinicians, who have used it in everyday clinical practice for decision‐making since ancient times.^33^ In contrast, appropriate statistical methods for this purpose have until recently been limited.^34^ The advent of so‐called joint models of longitudinal and time‐to‐event data greatly facilitates a systematic analysis of the prognostic relationship between a longitudinal risk factor trajectory and a clinical outcome.^14^, ^15^, ^16^ Our data demonstrate how useful these models are for dissecting complex risk factor–outcome relationship typically encountered in clinical cancer and thrombosis research. First, the model accounts for informative censoring. As reported in Table S2, a crude tabulation of D‐dimer values over time shows decreasing average D‐dimer levels over time. However, this is clearly an artefact, because patients with high D‐dimer develop VTE events and/or die,^19^ so that the remaining patient population becomes progressively “enriched” with low VTE‐risk patients and lower D‐dimer levels over time. The joint model accounts for this time‐dependent changes in the study population and thus reveals the true underlying biomarker trajectory in patients with and without VTE events. We can speculate that failure to account for this informative censoring explains why previous longitudinal biomarker analyses of cancer patients using simpler methods such as longitudinal box plots did not find consistent evidence for increasing D‐dimer levels in patients developing cancer‐associated VTE.^5^, ^18^ Second, the model cannot only estimate the association between a biomarker trajectory and clinical outcome, but also use this trajectory for providing dynamic time‐updated predictions for the individual patient. We could illustrate this prediction potential by using D‐dimer trajectories of individual patients to estimate their future risk of VTE. Conditional on the patients’ previous trajectories, predictions of 6‐month VTE incidence ranging from below 10% to above 20% were obtained. In the future, such prediction models may be implemented into online applications where physicians enter D‐dimer or clinical risk prediction model data into the application each time a patient comes for a clinical visit, and then obtain updated personalized predictions of thrombosis. We can speculate that these predictions may substantially improve the therapeutic ratio of prophylactic anticoagulation in the oncologic setting, but are also aware that the current study provides only a first proof‐of‐concept in this direction. Moreover, the joint model's association parameter for D‐dimer implies that D‐dimer can be used at any time of follow‐up (rather than at baseline) to predict risk of VTE. Considering that the risk of VTE is highest during the early treatment period, this finding could be interesting not only for initiating thromboprophylaxis, but also for terminating thromboprophylaxis once patients have survived beyond their personal “high VTE risk” period. Further, the D‐dimer trajectory plot in Figure 1 clearly shows that D‐dimer values can strongly vary within individual patients over his or her journey, depicting individuals with stable, decreasing, increasing, or grossly fluctuating biomarker levels. On one hand, this illustrates that considering hemostatic biomarkers in oncology as “static” variables is simplistic, and that a single biomarker marker measurement at a single point in time may only provide one “snapshot” of the underlying hypercoagulability. On the other hand, this finding supports the use of joint models for dynamic VTE risk assessment in the oncologic setting because these models can “update” a patient‐specific prediction even in the presence of highly fluctuating biomarker levels.

Seven limitations of our study should be discussed. First, although we analyzed one of the strongest known risk factors for cancer‐associated VTE (D‐Dimer), it is reasonable to assume that other static and dynamic factors such as type of chemotherapy, intercurrent infections or invasive diagnostic procedures, and cancer remission status will further modify VTE risk in a dynamic fashion.^35^, ^36^ Moreover, it can be reasonably speculated that these and potentially also other unmeasured risk factors may not only modify VTE risk but also the D‐dimer trajectory. Unfortunately, these time‐dependent data could not be included in the current analysis because of a lack of pertinent data. Nonetheless, joint models can easily accommodate multiple static and dynamic covariables into both the longitudinal and survival submodels of the joint model, which is of clear interest for both prediction and estimation purposes. Hence, future studies should aim to develop the joint modeling approach in this setting by examining the potential prognostic improvement in VTE risk assessment upon adding other risk factors to the models (“prediction”), or by quantifying how intermediate events such as treatment response and treatment failure modify the longitudinal biomarker trajectory (“estimation”). How joint models compare with other, potentially easier‐to‐apply methods in this setting, such as dynamic landmark analysis, could also be an interesting line of future investigation. Second, we did not consider mortality as a competing risk in the time‐to‐VTE component of the joint models, because competing risks within joint models are still an ongoing area of research and are not yet implemented in routine statistical analysis software.^26^ Rather, we used the Weibull model, which is a proportional hazards model with parametric representation of the baseline hazard. Not considering competing risks by using the Weibull model implies that the patient‐specific predictions of VTE risk may have been slightly overestimated (see Figure S2)^21^, ^22^, ^23^; more work will need to be done in the future to incorporate competing risks in this setting. Third, because of the rarity of prospective longitudinal biomarker studies in the oncologic setting.^5^ we could not externally validate our findings. Fourth, by design of the CATS study, we jointly analyzed several tumor entities. However, there is no a priori reason to assume that the association between the D‐dimer trajectory and VTE risk will be of similar magnitude and strength within individual tumor entities. Future studies may thus explore whether D‐dimer (or other biomarker or risk factor) trajectories have certain tumor‐specific relevance or irrelevance. Fifth, the sample size and absolute number of VTE events in our study was moderate, whereas the inclusion of predominantly “high‐VTE‐risk” tumor entities from our tertiary care university hospital such as primary brain tumors, lung cancer, and gastrointestinal cancers led to a cumulative VTE incidence of 12.1% that is much higher than usually observed in general oncology cohorts. For example, the cumulative 6‐month VTE incidences in the overall CATS study as well as in a global cohort of cancer patients undergoing chemotherapy (Multinational Cohort Study to Identify Cancer Patients at High Risk of Venous Thromboembolism) were only 5.7% and 6.3%, respectively.^4^ Thus, the statistical power supporting our results as well as the generalizability of our data across the entire cancer patient population must be interpreted within this limitation. Sixth, not only biomarkers, but also established VTE risk prediction models such as the Khorana score or the recently proposed CATS score, may be subject to longitudinal changes in cancer patients. Although a joint modeling analysis of these scores was considered beyond the scope of the present work, we think that it should be an important goal of future research to analyze whether their VTE risk stratification potential can be extended to the longitudinal setting. Finally, and most important, our study does not yet fully define how longitudinal D‐dimer measurements for dynamic reassessment of VTE risk could be best implemented in routine clinical practice to inform decisions about thromboprophylaxis. This pertains both to the timing of measurement, as well as to a potential longitudinal D‐dimer cutoff that would warrant the initiation of primary thromboprophylaxis. In our study, D‐dimer was measured each month, but whether this is feasible within a routine oncology setting needs to be further explored. We therefore encourage others to implement and validate the joint modeling process in patients with cancer for assessing VTE risk in external cohorts. In summary, we believe that the current study presents a first proof‐of‐concept rather than an immediately applicable system on how longitudinal biomarker trajectories can be used clinically for dynamically (re‐)assessing VTE risk in oncology.

## 5 CONCLUSION

In summary, we conclude that D‐dimer levels increase in patients with cancer before the onset of VTE, but remain stable in patients with cancer who do not develop VTE. The longitudinal trajectory of D‐dimer harbors important prognostic information on cancer‐associated VTE. Future studies should define how this concept may be best implemented in clinical practice for dynamically reassessing VTE risk in the oncologic setting.

## ADDENDUM

Conceived and designed the study: F. Posch, J. Riedl, EM Reitter, I. Pabinger, C. Ay. Collected data and contributed patients: F. Posch, J. Riedl, EM Reitter, E. Grilz. Performed laboratory analyses: P. Quehenberger. Performed statistical analyses: F. Posch. Contributed code for statistical analysis: MJ Crowther. Interpreted the results: all authors. Wrote the first draft of the manuscript: F. Posch. Revised the first draft of the manuscript: I. Pabinger, C. Ay. Contributed to the writing of the manuscript: all authors. Provided significant intellectual input to the revision of the manuscript: MJC. Agree with the manuscript's results and conclusions: all authors. ICMJE criteria for authorship read and met: all authors.

## CONFLICTS OF INTEREST

The authors do not declare any conflict of interest.

## ETHICAL APPROVAL

The ethical committee of the Medical University of Vienna (ethik‐kom@meduniwien.ac.at) approved the conduct of the project according to the revised version of the Declaration of Helsinki (EC no. 126/2003). All patients gave written informed consent before study inclusion.

## PRIOR PRESENTATION

Parts of this work were presented as an abstract at the December 2017 Annual Meeting of the American Society (ASH) in Atlanta, GA. The abstract was awarded an ASH Young Investigator Award to F.P.

## Supporting information

Supplementary Material
