# Supplementary material for: Dynamic assessment of venous thromboembolism risk in patients with cancer by longitudinal D‐Dimer analysis: A prospective study
Source: J Thromb Haemost. 2020 Apr 15;18(6):1348–56. doi: 10.1111/jth.14774 (PMC7317804; doi:10.1111/jth.14774)
Supplement: Supplementary file 1 — Supplementary Material [file JTH-18-1348-s001.pdf]

SUPPLEMENTARY DATA FILE

DYNAMIC ASSESSMENT OF VENOUS THROMBOEMBOLIC RISK IN PATIENTS WITH CANCER BY LONGITUDINAL D-DIMER ANALYSIS: A PROSPECTIVE STUDY.

Running head: D-Dimer trajectories in cancer-associated thrombosis

Florian Posch, MD MSc,[1,2,3] Julia Riedl, MD PhD,[1] Eva-Maria Reitter, MD PhD,[1] Michael J. Crowther, PhD,[4] Ella Grilz, MD,[1,5] Peter Quehenberger, MD,[6] Bernd Jilma, MD,[7] Ingrid Pabinger, MD[1], and Cihan Ay, MD[1,8]

[1]Clinical Division of Haematology and Haemostaseology; Department of Medicine I; Medical University of Vienna; Währinger Gürtel 18-20; A-1090 Vienna; Austria

[2]Division of Oncology; Department of Internal Medicine; Medical University of Graz; Auenbruggerplatz 15; A-8036 Graz; Austria

[3]Center for Biomarker Research in Medicine (CBmed Ges.m.b.H.); Stiftingtalstrasse 5; A-8010 Graz; Austria

[4]Centre for Medicine; Department of Health Sciences; University of Leicester; Leicester, LE1 7RH; United Kingdom

[5]Department of Anaesthesia and Critical Care; SMZ Ost – Danube Hospital; Langobardenstrasse 122; A-1220 Vienna; Austria

[6]Department of Laboratory Medicine; Medical University of Vienna; Währinger Gürtel 18-20; A-1090 Vienna; Austria

[7]Section of Hematology & Immunology; Department of Clinical Pharmacology; Medical University of Vienna; Währinger Gürtel 18-20; A-1090 Vienna; Austria

[8]I.M. Sechenov Fist Moscow State Medical University (Sechenov University); Moscow; Russia

Supplementary Tables

Supplementary Table 1

**Variable**	HR	95%CI	p
Age at entry (per 5 years increase)	1.00	0.84-1.20	0.974
Female Gender	1.36	0.57-3.26	0.494
BMI (per 5kg/m² increase)	1.61	1.11-2.31	**0.011**
Newly-diagnosed malignancy	1.03	0.14-7.71	0.976
Tumor stage*	/	/	/
---Local (TNM N0 M0)	Ref.	Ref.	Ref.
---Locally-advanced (TNM N+ M0)	1.00	0.28-3.56	0.996
---Metastatic (TNM M1)	1.34	0.49-3.68	0.573
Tumor site**	/	/	/
---Low/Moderate VTE risk tumor sites	Ref.	Ref.	Ref.
---High VTE risk tumor sites	0.61	0.10-3.66	0.591
---Very high VTE risk tumor sites	2.29	0.52-10.02	0.271
Khorana score (per 1 point increase)	1.22	0.72-1.75	0.607
Khorana score high (≥3 points)	0.77	0.23-2.64	0.680
Khorana score high (≥2 points)	1.99	0.72-5.47	0.184
Haemoglobin < 10g/dL and/or ESA use	N/E	N/E	N/E
White blood count ≥ 11G/L	0.65	0.15-2.79	0.559
Platelet count ≥ 350G/L	N/E	N/E	N/E
BMI ≥ 35kg/m²	3.44	0.46-25.72	0.229
D-Dimer at baseline (per doubling)	1.73	1.32-2.27	**<0.0001**

Supplementary Table 2

Visit	Number of patients / Number of patients with D-Dimer available	D-Dimer in µg/mL (median [25th-75th percentile])
Baseline visit	167 / 165	0.97 [0.54-2.05]
Follow-up visit #1	155 / 152	0.90 [0.41-1.91]
Follow-up visit #2	137 / 137	0.82 [0.43-2.36]
Follow-up visit #3	106 / 105	0.90 [0.41-1.56]
Follow-up visit #4	85 / 83	0.61 [0.41-1.56]
Follow-up visit #5	68 / 68	0.63 [0.40-1.87]
Follow-up visit #6	51 / 51	0.57 [0.33-1.31]

Supplementary Table 3

Component	Variable	Interpretation of coefficient	Coefficient	95%CI	p
Longitudinal component	Change in D-Dimer (µg/mL/month)	Change in D-Dimer over time (µg/mL/month)	-0.08	-0.18-0.02	0.140
	Change in D-Dimer # Metastatic disease	Change of the change in D-Dimer over time for having metastatic disease (µg/mL/month)	0.12	-0.01-24.9	0.080
	Constant	Mean D-Dimer at baseline	1.85	1.46-2.23	<0.0001
Time-to-VTE component	Metastatic disease	Relative change in the hazard of VTE for patients with metastatic disease (i.e. hazard ratio)	1.54	0.29-8.15	0.615
Association parameter α	D-Dimer trajectory (per 1µg/mL increase) in patients without metastatic disease	Relative change in the hazard of VTE per 1µg/mL increase of the D-Dimer trajectory (i.e. hazard ratio) in patients without metastatic disease	1.90	1.14-3.18	0.014
	D-Dimer trajectory (per 1µg/mL increase) in patients with metastatic disease	Relative change in the hazard of VTE per 1µg/mL increase of the D-Dimer trajectory (i.e. hazard ratio) in patients with metastatic disease	1.46	1.20-1.77	<0.0001

Supplementary Table 4

Component	Variable	Interpretation of coefficient	Coefficient	95%CI	p
Longitudinal component	D-Dimer trajectory (µg/mL/month)	Change in D-Dimer over time in µg/mL/month	-0.01	-0.16-0.14	0.855
	Khorana score # D-Dimer trajectory	Change of the change in D-Dimer over time in µg/mL/month per 1 point increase in the baseline Khorana score	-0.01	-0.08-0.06	0.838
	Constant	Mean baseline D-Dimer	1.84	1.45-2.23	<0.0001
Time-to-VTE component	Khorana score (per 1 point increase)	Relative change in the hazard of VTE per 1 point increase in the baseline Khorana score (i.e. hazard ratio)	1.09	0.71-1.68	0.691
Association parameter α	D-Dimer trajectory	Relative change in the hazard of VTE per 1µg/mL increase in the D-Dimer trajectory (i.e. hazard ratio)	1.45	1.25-1.69	<0.0001

Supplementary Table legends

Supplementary Table 1. Univariable baseline predictors of VTE risk. All regression results were obtained with univariable Weibull regression models. P-values ≤0.05 are reported in **bold** font. *Patients with primary brain tumors were assigned to the "local" stage group. **Tumor site categories were defined as in the original publication by Khorana et al. (i.e. colorectal cancer included in the "low/moderate VTE risk" group),[1] with brain tumors being assigned to the "very high VTE risk" group according to Ay C et al.[2] Abbreviations: 95%CI: 95% confidence interval, p – Wald test p-value, BMI – Body Mass Index, TNM – Tumor Node Metastasis classification, VTE – Venous thromboembolism, ESA – Erythropoiesis-stimulating agents. N/E – not estimable (no VTE events were observed during the 250-day follow-up period among the n=2 patients with Haemoglobin< 10g/dL and/or ESA use and among the n=29 patients with Platelet count ≥ 350G/L).

Supplementary Table 2. Evolution of D-Dimer and the patient population across follow-up visits. The tables shows decreasing average D-Dimer levels over time, consistent with a "removal" of patients with high D-Dimer levels from the at-risk population (explained by the strong impact of D-Dimer on VTE and mortality).[3]

Supplementary Table 3. A multivariable joint model of the D-Dimer trajectory and prospective thrombotic risk – Adjusted for metastatic disease status at baseline. An increased D-Dimer trajectory predicted for higher VTE risk in both patients with and without metastatic disease at baseline, although the magnitude of association was slightly smaller in patients with metastatic disease. For ease of interpretation, the meaning of all regression

coefficients is explained in the column “Interpretation of coefficient.” # denotes an interaction. Abbreviations: 95%CI – 95% confidence interval, p – Wald-test p-value.

Supplementary Table 4. A multivariable joint model of the D-Dimer trajectory and prospective risk of VTE adjusted for the baseline Khorana score. The association between a higher D-Dimer trajectory and a higher risk of VTE prevailed also after adjusting for the baseline Khorana score. The baseline Khorana score was not associated with the D-Dimer trajectory (i.e. patients with higher Khorana scores had similar D-Dimer trajectories as patients with lower Khorana Scores), and did not predict the occurrence of VTE (see also Supplementary Table 1). For ease of interpretation, the meaning of all regression coefficients is explained in the column “Interpretation of coefficient.” # denotes an interaction. Abbreviations: 95%CI – 95% confidence interval, p – Wald-test p-value, VTE – Venous thromboembolism.

Supplementary Figures

Supplementary Figure 1

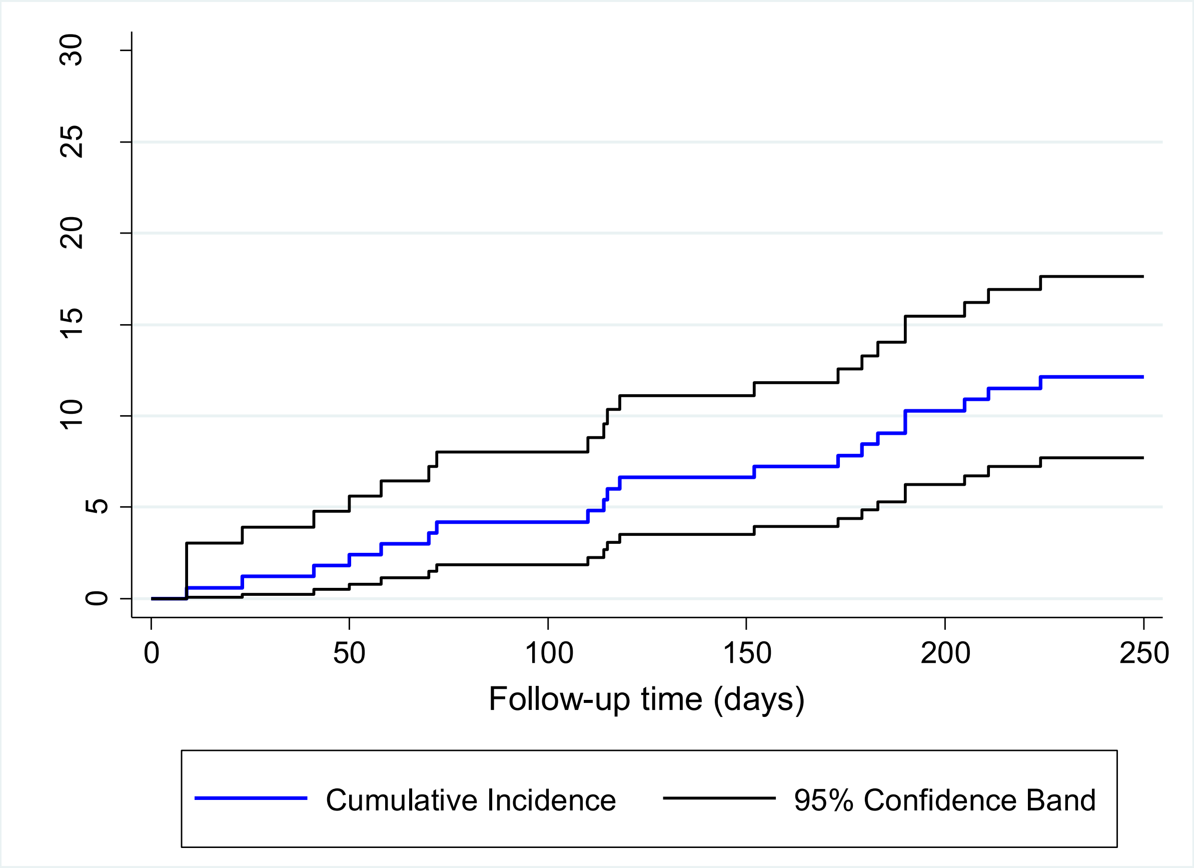

Supplementary Figure 2

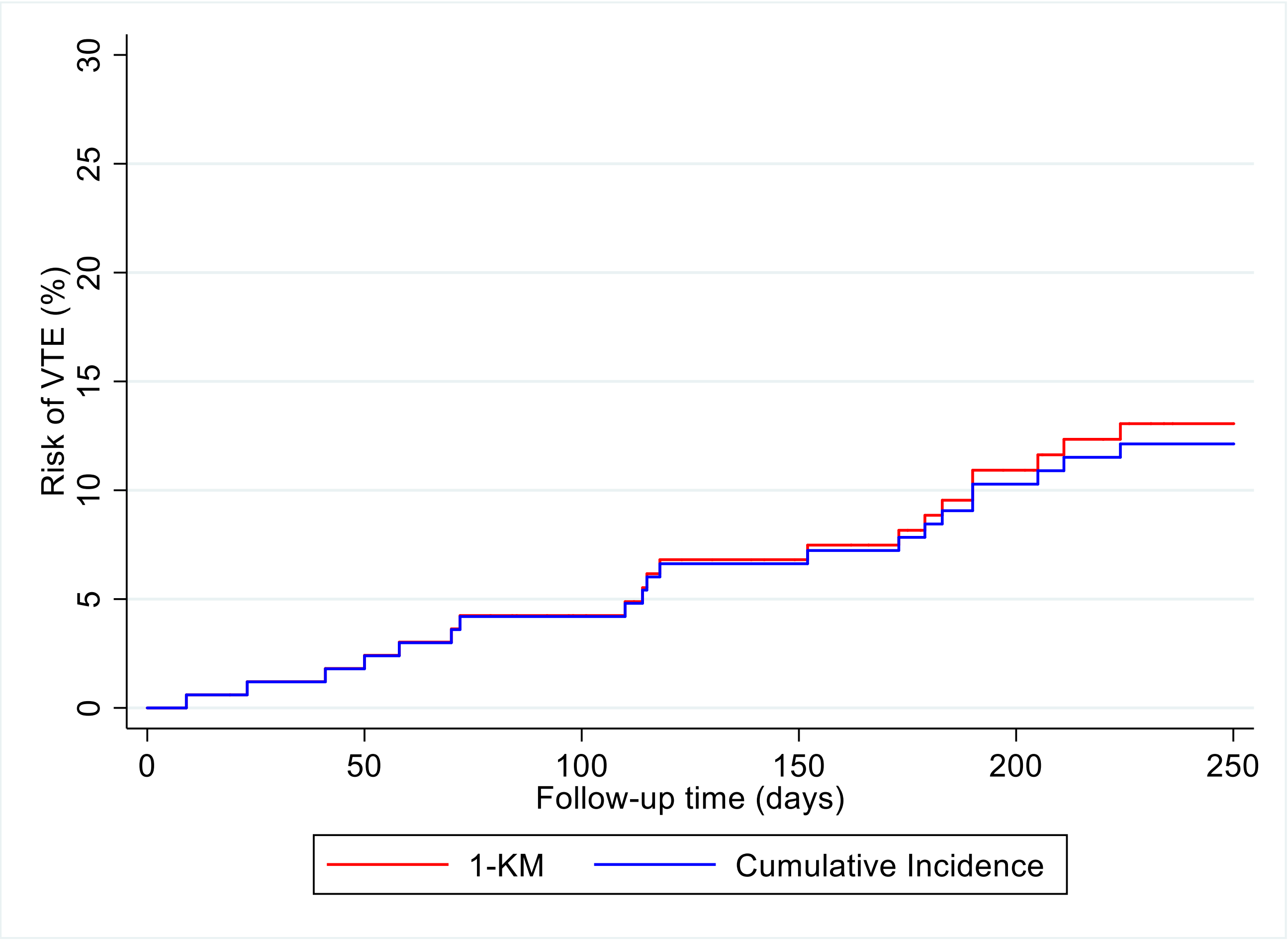

Supplementary Figure legends

Supplementary Figure 1. Cumulative incidence of VTE during the 250-day study period (n=167, 20 VTE events). VTE risk was estimated with a competing risk cumulative incidence estimator, accounting for death-from-any-cause-other-than-fatal-VTE as the competing event of interest. Dashed blue lines represent 95% confidence bands. Abbreviations: VTE – Venous Thromboembolism.

Supplementary Figure 2. Overestimation of 250-day VTE risk with the 1-Kaplan-Meier approach as compared to a competing risk approach (n=167, 20 VTE events). VTE risk was estimated with a 1-Kaplan-Meier estimator (red line), and a competing risk cumulative incidence estimator, accounting for death-from-any-cause-other-than-fatal-VTE as the competing event of interest (blue line). Abbreviations: VTE – Venous Thromboembolism, 1-KM – 1-Kaplan-Meier estimator.
